# Jestings

**Published:** 1879-02

**Authors:** 


—"I've got a new machine," said a Yankee pedler, "for picking bones out of fishes. Now I tell you, its a leetle bit the queerest thing you ever did see. All you have to do is to set it on a table and turn a crank, and the fish flies down your throat, and the bones rite under the grate. Well, there was a country greenhorn got hold of it the other day, and he turned the crank the wrong way; and, I tell you, the way the bones flew down his throat was awful! Why, it stuck the fellow so full of bones that he couldn't get his shirt off for a whole week."

—A young couple had been married by a Quaker, and after the ceremony, he remarked to the husband: "Friend thou art at the end of thy troubles." A few weeks after, the young man came to the good minister boiling over with rage, (his wife was a regular vixen.) "I thought you told me that I was at the end of my troubles?" "So I did, friend, but I did not say which end."

—The report of a court-martial was sent to President Lincoln for his examination, who returned it with this characteristic endorsement: "Whereas, F. W. Smith had transaction with the U. S. Navy Department to a million and a quarter of dollars, and had a chance to steal a quarter of a million; and whereas he was charged with stealing only ten thousand dollars, and from the final revision of the testimony it is only claimed that he stole one hundred dollars, I don't believe that he stole anything at all. Therefore the records of the court-martial, together with the findings and sentence, are disapproved, declared null and void, and the defendant is fully discharged. (Signed) A. LINCOLN."

—An impatient Welshman called to his wife, "Come, come, isn't breakfast ready? I've had nothing since yesterday, and to-morrow will be the third day!" This is equal to the call of the stirring housewife, who aroused her maid at four o'clock with, "Come, Bridget, get up! Here, 'tis Monday morning, to-morrow is Tuesday, the next day's Wednesday—half the week gone, and nothing done yet!"

—A neighbor was telling me of a little boy whose father was a great church-goer, and reverenced the sanctuary. He had been very naughty, and his mother had told him that she was going to tell his father. He quietly retired to the barn, where there was a paint-pot and brush, and painted a church on the seat of his pants, and running to his mother, he says: "O mother! I guess father will not whip me, for I have painted a church on my pantaloons, and he will not whip the church."

—The alderman who was lately injured by the accidental discharge of his duty, is reported to be in a fair way of recovery. He says he'll never be caught that way again while in the full possession of his senses.

—A fashionable party is now called a "daughtercultural show."

—"They say 'cotton is declining,'" exclaimed an old lady, as she removed her spectacles and laid down her paper. "I thought so," she continued, "for the last thread I used was very feeble."

—"I am glad this coffee don't owe me anything," said a bookkeeper to his wife the other morning at breakfast. "Why so?" was the question. "Because I don't believe it would ever settle."

—HIBERNIAN TOASTS.—Two gallant "sons of Erin," being just discharged from service, were rejoicing over the event, when one, who felt all the glory of his own noble race, suddenly raised his pot above his head, and said, "Arrah, Mike, here's to the gallant old 69th—the last in the field, and the first to leave it." "Tut, tut, man," said Mike; "ye don't mane that." "Don't mane it, is it?" Then what do I mane?" "You mane," said Mike, and he raised his glass high, and looked lovingly at it, "Here's to the gallant 69th, equal to none!" And so they drank.

—A learned and enthusiastic orator recetly startled his audience by the following sentence: "Sir, let those beware who would trifle with the popular will. In the language of the poet, '*Facilis descensus averni*'—The voice of the people is the voice of God." This is nearly as good as the illustration once used by a member of a certain Down East Legislature: "Mr. Speaker," said the member, referring to the question under debate, "this matter is like Pandora's box—the more you stir it, the worse it smells!"

—A youth, who much desired to wear the matrimonial yoke, had not sufficient courage to pop the question. On informing his father of the difficulty he labored under, the old gentleman replied, passionately, "Why, you great booby, how do you suppose I managed when I got married?" "Oh, yes, you married Mother, but I've got to marry a strange girl!" said the bashful lover.

—How many rods make an acre?" a father asked of his son, a fast urchin, as he came home one night from a town school. "Well, I don't know, governor," was the reply of the young hopeful, "but I guess you'd think one rod made an acre, if you'd got such a tanning as I did from old vinegar face this afternoon."

—The owner of a large dog at Grand Rapids, Mich., a few days ago, placed a hundred dollar looking-glass before his canine to worry him. The dog flew round barking and growling. The owner was delighted and cried "seek 'em!" The dog "seeked," and the mirror and the "other dog" disappeared at at the same time.

—The editor of *The Woonsocket Patriot* makes merry over the mistake of an old Shanghai hen of his that has been "setting" for five weeks upon two round stones and a brick! "Her anxiety," quote he, "is no greater than ours to know what she will hatch. If it proves a brickyard that hen is not for sale.

—A young lady went into a store to buy a piece of music, entitled, "When I sleep I dream of thee; but by a ludicrous mistake, she astonished the young man by inquiring if he had the music entitled, "When I dream I sleep with thee."

—A down-east Yankee, seeing an alligator for the first time on the Mississippi, with his mouth open, he exclaimed: "Well, he ain't what you may call a hansum critter. But he's got a good deal of openness when he smiles.

—A man out West says he moved so many times during one year that whenever a covered wagon stopped at the gate his chickens would fall on their backs and hold up their feet, in order to be tied and thrown in.

—A Shilling Temptation.—A young man in England, having entertained a tender passion for a young woman, felt such insurmountable diffidence as to prevent his ever disclosing the same to the fair empress of his heart, and resolved on an expedient which would bring the business to an issue. He went to the clergyman, and requested the bans of marriage might be published according to law. When the publication was brought to her ears, she was filled with astonishment, and went to him to vent her resentment; he bore the sally with fortitude, observing that if she did not think proper to have him, she could go to the clergyman and forbid the bans. After a moment's pause, she took counsel with her anger, and said: "Well, as it has been dohe, it is a pity that the shilling should be thrown away!

—A little ragged urchin, begging in the city the other day was asked by a lady who filled his basket, if his parents were living? "Only dad, marm," said the boy. "Then you have enough in your basket now to feed the family for some time," said the lady. "Oh, no, I haven't neither," said the lad, "for dad and me keeps five boarders; he does the house work, and I do the market'n."

—Never Mind the Wood Shed.—"My dear Amelia," said a dandy, "I have long wished for this opportunity, but hardly dare speak now for fear you will reject me. But I love you; say you will be mine! Your smiles would shed—" and then he came to a pause; "your smiles would shed—" and then he paused again. "Never mind the wood shed," said Amelia, "go on with the pretty talk."

—A young Miss who was visiting in a neighboring town recently received a letter from a friend at home, in reply to one which she had made a request to be told "all the news." Her friend wrote: "I have not much to write to you, but the ladies' aid society meets at our house next week, when I shall hear the news about everbody in town."

—Which has most legs, a horse or no horse? No horse has five.

—If a toper and a gallon of whisky were left together, which would be drunk first?

—When do you indulgein your most extravagant repast? When you have a piano for tea (forte).

—Why is a heartless kiss like a stage on a cold day? Because it's a 'bus with no warmth in it.

—A gentleman, one evening, was seated near a lovely woman, when the company around were proposing conundrums to each other. Turning to his companion, he said, " Why is a lady unlike a mirror? She " gave up." "Because," said the rude fellow, " a mirror reflects without speaking; a lady speaks without reflecting." " Very good," said she. " Now answer me. Why is a man unlike a mirror?" " I cannot tell you." " Because the mirror is polished, and the man is not."

—The man who takes things easy---the pickpocket.

---A soldier with a huge pair of understandings called upon a boot black the other day to shine up his boots. The urchin contemplated the size of the job for a moment, and then called out to a comrade, " I say, Bill, lend us a spit, won't yer? I've got an army contract."

---" I'll bet a sheep," said old Meredith to his better half, " that our boy Otho is goin' crazy: fur he's grinnin' at the plow, he's grinnin' at the barn and he's grinnin' to himself wherever he goes." "Sho! old man," said his wife, "you don't know nothin." The critter's got a love letter."

---A little shaver was sent to an undertaker, to order a coffin for an infant. "How old was the child," asked the undertaker. " It was not old at all," was the answer, " it died a bornin." This " plan" of the reconstruction committee has met the same untimely fate.

---Sensible Advice.---An exchange paper among other suggestions which will enable a person to avoid the cholera, says: " Endeavor, if possible, to keep a clean conscience, and two or three clean shirts. Rise with the lark, but avoid larks in the evening. Be above ground in all your dwellings, and above board in all your dealings. Love your neighbor as yourself, but don't have too many in the same house with you.

----We trust the Lord is on our side, Mr. Lincoln," said the speaker of a delegation of Christian men to that good man, during one of the darkest days of the rebellion. " I do not regard that so essential as something else," replied Mr. Lincoln. The pious visitor looked horror struck until the President added: " I am most concerned to know that we are on the Lord's side.'

